# AaWRKY17, a positive regulator of artemisinin biosynthesis, is involved in resistance to *Pseudomonas syringae* in *Artemisia annua*

**DOI:** 10.1038/s41438-021-00652-6

**Published:** 2021-10-01

**Authors:** Tiantian Chen, Yongpeng Li, Lihui Xie, Xiaolong Hao, Hang Liu, Wei Qin, Chen Wang, Xin Yan, Kuanyu Wu-Zhang, Xinghao Yao, Bowen Peng, Yaojie Zhang, Xueqing Fu, Ling Li, Kexuan Tang

**Affiliations:** 1 Joint International Research Laboratory of Metabolic and Developmental Sciences, Frontiers Science Center for Transformative Molecules, Plant Biotechnology Research Center, Fudan-SJTU-Nottingham Plant Biotechnology R&D Center, School of Agriculture and Biology, Shanghai Jiao Tong University, Shanghai, 200240 China; 2 Laboratory of Medicinal Plant Biotechnology, College of Pharmacy, Zhejiang Chinese Medical University, Hangzhou, 310053 China

**Keywords:** Plant molecular biology, Transcriptional regulatory elements

## Abstract

*Artemisia annua*, a traditional Chinese medicinal plant, remains the only plant source for artemisinin production, yet few genes have been identified to be involved in both the response to biotic stresses, such as pathogens, and artemisinin biosynthesis. Here, we isolated and identified the WRKY transcription factor (TF) AaWRKY17, which could significantly increase the artemisinin content and resistance to *Pseudomonas syringae* in *A. annua*. Yeast one-hybrid (Y1H), dual-luciferase (dual-LUC), and electrophoretic mobility shift assay (EMSA) results showed that AaWRKY17 directly bound to the W-box motifs in the promoter region of the artemisinin biosynthetic pathway gene *amorpha-4,11-diene synthase* (*ADS*) and promoted its expression. Real-time quantitative PCR (RT-qPCR) analysis revealed that the transcript levels of two defense marker genes, *Pathogenesis-Related 5* (*PR5*) and *NDR1/HIN1-LIKE 10* (*NHL10*), were greatly increased in *AaWRKY17-*overexpressing transgenic *A. annua* plants. Additionally, overexpression of *AaWRKY17* in *A. annua* resulted in decreased susceptibility to *P. syringae*. These results indicated that AaWRKY17 acted as a positive regulator in response to *P. syringae* infection. Together, our findings demonstrated that the novel WRKY transcription factor AaWRKY17 could potentially be used in transgenic breeding to improve the content of artemisinin and pathogen tolerance in *A. annua*.

## Introduction

Plants are exposed to various stresses from the environment during their lifecycle, including abiotic stresses such as drought and biotic stresses such as pathogens^1,2^. For survival and the continuation of the next generation, plants have adopted many defense mechanisms against biotic and abiotic stresses^3^. Among the various biotic stresses, pathogens are considered major threats to plant growth, development, and yield. To cope with pathogenic stress, plants have developed sophisticated innate immunity pathways, including pathogen-associated molecular pattern-triggered immunity and effector-triggered immunity, which help them avoid greater pathogenic invasion^4–6^.

The WRKY family forms a transcriptional network that regulates the complex signaling network in the plant defense system against pathogen infection^7^. As one of the largest TF families in plants, WRKY proteins are identified by two highly conserved domains: the amino acid motif WRKYGQK at the N-terminus and a C_2_H_2_ or C_2_HC zinc-finger motif at the C-terminus. Moreover, WRKY TFs can regulate the expression level of downstream target genes by directly binding to W-boxes (TTGAC(C/T)) in their promoter regions^8^. Extensive research has shown that WRKY TFs play key roles in plant resistance to several pathogenic bacterial species^9^. In plants, *Pseudomonas syringae*^10^, *Botrytis cinerea*^11^, *Sclerotinia sclerotiorum*^12^, and *Ralstonia solanacearum*^13^ are the most common pathogens and have been well studied over the past decades. It is worth noting that many WRKY genes have been shown to enhance resistance to multiple pathogens. For example, *Arabidopsis thaliana* WRKY8, WRKY3, and WRKY4 enhanced resistance to *P. syringae* and *B. cinerea* infection^14,15^.

Artemisinin, a sesquiterpene lactone endoperoxide, is specifically synthesized in glandular trichomes^16,17^ and isolated from the traditional Chinese medicinal plant *A. annua*. It is well known that artemisinin is the best therapeutic agent against malaria and is also effective in the treatment of several cancers^18–20^. The artemisinin biosynthetic pathway has been extensively elucidated, and *amorpha-4,11-diene synthase* (*ADS*), *Cyt P450-dependent hydroxylase (CYP71AV1)*, *artemisinic aldehyde _11(13) reductase* (*DBR2*), and *aldehyde dehydrogenase 1* (*ALDH1*) are the key genes that encode enzymes catalyzing artemisinin production^21^. In addition to these structural genes, several lines of evidence have suggested that TFs play pivotal roles in artemisinin biosynthesis^22–24^. However, limited information is available about the role of *A. annua* TFs in biotic stress defense. Currently, *A. annua* is the only plant source for artemisinin production. However, the artemisinin content in wild-type (WT) *A. annua* was low. Therefore, it is important to develop *A. annua* germplasms with high yield and quality. Several studies have indicated that WRKY family members are involved in artemisinin production. For instance, AaWRKY1 is a positive regulator of *ADS* and *CYP71AV1*^25^. AaGSW1 (GLANDULAR TRICHOME-SPECIFIC WRKY 1) was reported to increase artemisinin biosynthesis by directly binding to the promoter of *CYP71AV1*^26^. However, few researchers have been able to identify any WRKY TFs that are involved in the regulation of plant resistance to some biotic stresses in *A. annua*.

In this study, we isolated a new WRKY TF, AaWRKY17, which positively regulates the transcription of *ADS* by directly binding to its W-box motifs in the promoter, thus resulting in an increased artemisinin content. In addition, we demonstrated that the expression of *AaWRKY17* was significantly induced by treatment with salicylic acid (SA), methyl jasmonate (MeJA), and *P. syringae* infection. Many phytohormones, such as SA, JA^27^, ABA, and ET^28,29^, can mediate the signaling pathways of plant responses to biotic stresses, and the expression of WRKY genes during plant defense responses also has a close relationship with these signaling pathways^30^. AtWRKY28 and AtWRKY75 mainly activate the JA/ET pathway to defend *A. thaliana* against *S. sclerotiorum*^12^. AtWRKY18, through activating *PR* gene expression in the SA pathway^31^, enhances the resistance of *A. thaliana* to *P. syringae*. Moreover, we showed that overexpression of *AaWRKY17* significantly increased the transcript levels of the defense marker genes *PR5*^27,32^ and *NHL10*^33^ and enhanced the resistance of *A. annua* to *P. syringae*. In conclusion, our research reveals a novel WRKY TF with dual functions in both artemisinin biosynthesis and biotic stress defense in *A. annua*, which can potentially be used in developing high-yielding and pathogen-resistant *A. annua*.

## Results

### Cloning and characterization of *AaWRKY17*

**Fig. 1:**
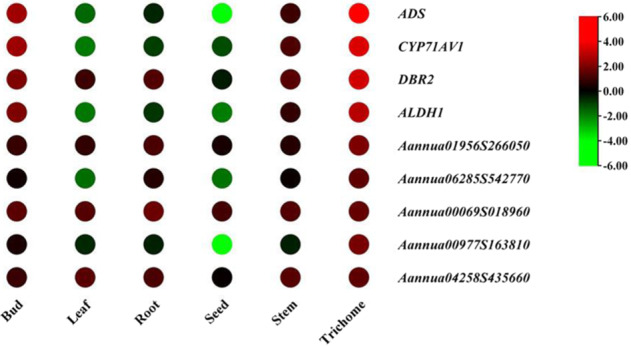
Global expression profile of five candidate genes. Heatmap of the expression levels of five candidate genes and *ADS, CYP71AV1, DBR2*, and *ALDH1* in different tissues

**Fig. 2:**
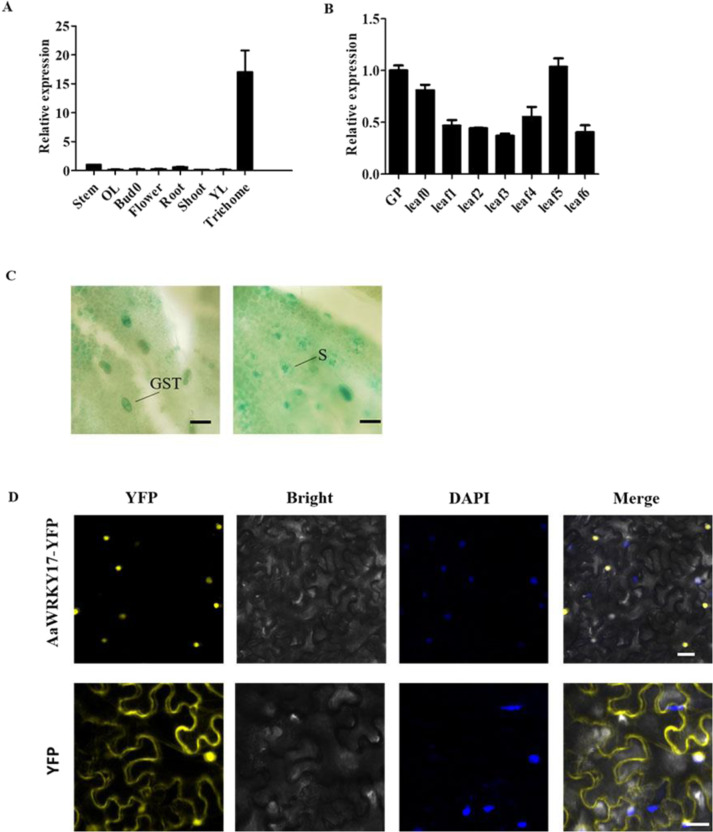
Characterization of *AaWRKY17*. **A** The expression level of *AaWRKY17* in different tissues of *A. annua*. **B** The expression level of *AaWRKY17* at different positions of *A. annua* leaves. **A**, **B** were measured by RT-qPCR, and actin was used as an internal reference. Data are given as the mean ± SD (*n* = 3). **C** *AaWRKY17* promoter-GUS (*pAaWRKY17*-GUS) expression in the young leaves of *A. annua*. GST glandular secreting trichome, S stoma. Bars: 20 μm. **D** Subcellular localization of 35S:AaWRKY17-YFP in tobacco leaf epidermal cells. Yellow, yellow fluorescent protein (YFP). Blue, 4′, 6-diamidino-2-phenylindole staining (DAPI). Bars: 30 μm

There are 135 WRKY genes in *A. annua*. Based on the transcriptome data, five WRKY TFs with high RPKM (reads per kilobase per million reads) values in trichomes were selected (Fig. 1): *Aannua00069S018960*, *Aannua00977S163810*, *Aannua04258S435660*, *Aannua06285S542770*, and *Aannua01956S266050*. RT-qPCR results of different tissues showed that *Aannua01956S266050* exhibited the highest expression level in trichomes among the five candidate genes, which was consistent with the transcriptome data from different *A. annua* tissues (Fig. 2A and Supplementary Fig. S1). Since artemisinin is only produced in trichomes of *A. annua*, we speculated that *Aannua01956S266050* potentially participated in artemisinin biosynthesis. We named the gene *Aannua01956S266050 AaWRKY17* for further study according to the close relationship with *AtWRKY17* in *A. thaliana* (Supplementary Fig. S2).

### Expression pattern and subcellular localization of AaWRKY17

The expression pattern of *AaWRKY17* in different tissues showed that it was highly expressed in trichomes (Fig. 2A). The expression profiling of *AaWRKY17* in leaves at different positions showed that *AaWRKY17* exhibited a stable expression level during the development of *A. annua* leaves (Fig. 2B). To further elucidate the expression pattern of *AaWRKY17*, we conducted *AaWRKY17* promoter-driven β-glucuronidase (GUS) transformation of *A. annua*. We observed that GUS was strongly detected in the glandular trichomes and stomas (Fig. 2C). To investigate the subcellular localization of AaWRKY17 in vivo. The AaWRKY17 protein was fused with the Yellow fluorescent protein (YFP) protein and transiently expressed in tobacco leaves. YFP fluorescence was detected in the nucleus (Fig. 2D), which indicated that AaWRKY17 might function as a transcription factor.

### Overexpression of *AaWRKY17* in *A. annua* increases artemisinin biosynthesis, and downregulation of *AaWRKY17* decreases artemisinin biosynthesis

**Fig. 3:**
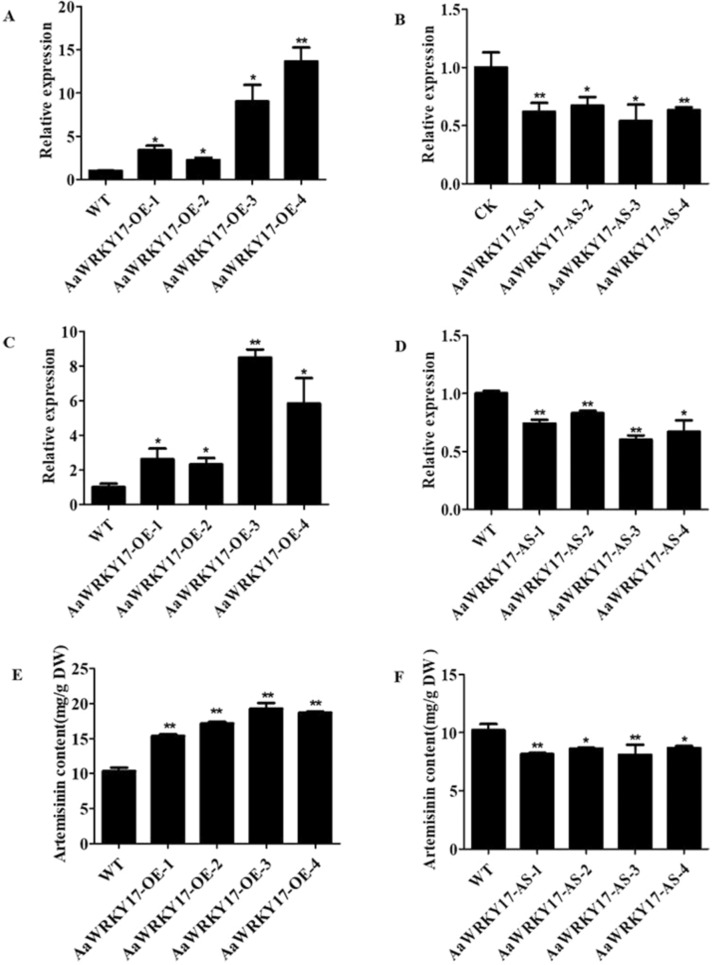
Analysis of *AaWRKY17* transgenic *A. annua* plants. Expression levels of *AaWRKY17* in different *AaWRKY17* overexpression (**A**) and antisense lines (**B**). Transgenic plants were measured by RT-qPCR. WT, wild-type. Actin was used as an internal reference. Expression levels of *ADS* in different *AaWRKY17* overexpression (**C**) and antisense lines (**D**). Transgenic plants were measured by RT-qPCR. WT, wild-type. Actin was used as an internal reference. Artemisinin content in different *AaWRKY17* overexpression (**E**) and antisense lines (**F**), measured by high-performance liquid chromatography (HPLC). All data are given as the means ± SD (*n* = 3) *p < 0.05; **p < 0.01; Student’s *t* test

To test whether AaWRKY17 is a regulator of artemisinin biosynthesis, we overexpressed *AaWRKY17* in *A. annua* and selected the transgenic lines AaWRKY17-OE-1, AaWRKY17-OE-2, AaWRKY17-OE-3, and AaWRKY17-OE-4 for further study according to their high expression levels of *AaWRKY17*. Compared with the WT plants, the transcript level of *AaWRKY17* was increased 2.3- to 13.6-fold in the transgenic lines AaWRKY17-OE-1, AaWRKY17-OE-2, AaWRKY17-OE-3, and AaWRKY17-OE-4 (Fig. 3A). The leaves of 5-month-old *AaWRKY17-*overexpressing transgenic plants were collected to measure artemisinin content using high-performance liquid chromatography (HPLC). In *AaWRKY17*-overexpressing lines, the artemisinin contents were significantly increased by 49.5–87.4% (Fig. 3E). To further prove the function of *AaWRKY17* in the regulation of artemisinin biosynthesis in *A. annua*, we downregulated its expression by the RNA antisense approach. The transcript level of *AaWRKY17* was suppressed to 54–68% of the WT plant level in the selected *AaWRKY17* antisense transgenic plants AaWRKY17-AS-1, AaWRKY17-AS-2, AaWRKY17-AS-3, and AaWRKY17-AS-4 (Fig. 3B). Compared with the WT plants, the contents of artemisinin in *AaWRKY17* antisense lines were decreased by 14.7–20.6% (Fig. 3F). In addition, we detected the transcript levels of four key enzyme genes of the artemisinin biosynthetic pathway, including *ADS*, *CYP71AV1*, *DBR2*, and *ALDH1*. The expression level of *ADS* was increased 2.3- to 8.5-fold in the *AaWRKY17*-overexpressing transgenic lines AaWRKY17-OE-1, AaWRKY17-OE-2, AaWRKY17-OE-3, and AaWRKY17-OE-4 (Fig. 3C). In the *AaWRKY17* antisense transgenic lines AaWRKY17-AS-1, AaWRKY17-AS-2, AaWRKY17-AS-3, and AaWRKY17-AS-4, the transcript level of *ADS* was suppressed to 60–83% of the control level (Fig. 3D). However, no visible expression changes in *CYP71AV1*, *DBR2*, and *ALDH1* were observed in either *AaWRKY17-*overexpressing or *AaWRKY17* antisense lines. These results indicated that AaWRKY17 might be a positive regulator of artemisinin biosynthesis by activating the expression of *ADS*.

### AaWRKY17 directly binds to and activates the promoter of *ADS*

**Fig. 4:**
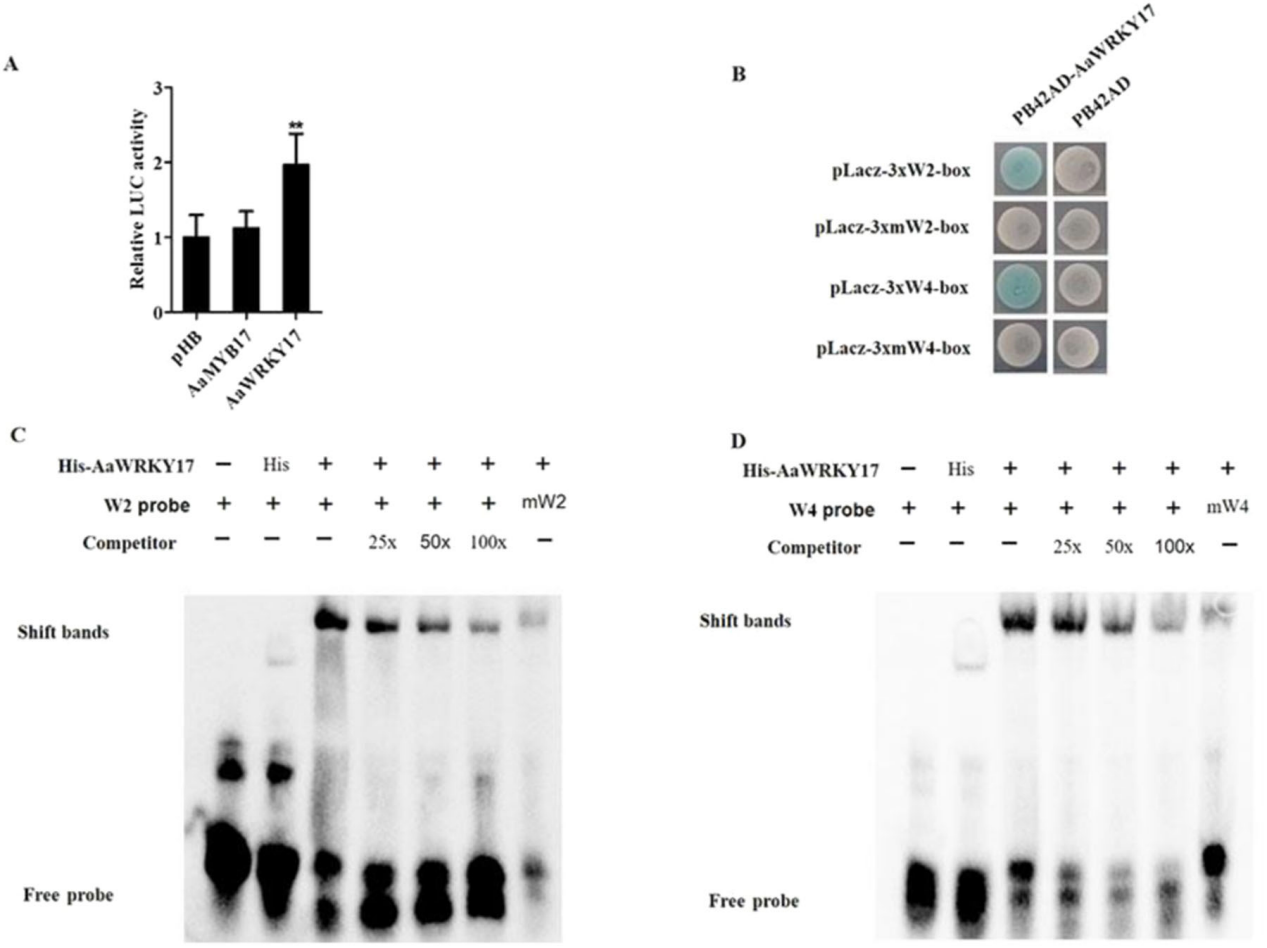
AaWRKY17 directly binds to and activates the promoter of *ADS*. **A** Effects of AaWRKY17 on *ADS* promoter activation. The activity of the *ADS* promoter fused to the LUC reporter was determined using a transient dual-LUC assay in tobacco. The relative LUC activity was normalized to the reference Renilla (REN) luciferase. Data are given as means ± SD (*n* = 4). Student’s *t* test: *p < 0.05, **p < 0.01. The empty vector pHB was used as a negative control. The vector pHB-AaMYB17 was used as a positive control. **B** Yeast one-hybrid assay of protein–DNA interactions. The empty vector pB42AD was used as a negative control. **C**, **D** Electrophoretic mobility shift assay (EMSA) showing AaWRKY17 binding to the W2 box (−2174 to −2179) and W4 box (−298 to −303) of the *ADS* promoter. Purified His-AaWRKY17 fusion protein and biotin-labeled probes were used. Nonlabeled probes (25x, 50x, 100x) were added as competitors. Purified His-pCold proteins were used as a negative control

Since *AaWRKY17-*overexpressing lines showed increased expression of *ADS*, we next investigated the regulatory relationship between AaWRKY17 and *ADS*. Dual-luciferase (dual-LUC) assay results showed that AaWRKY17 could significantly activate the *ADS* promoter (Fig. 4A). Previous studies have reported that WRKY domains can specifically bind to W-box sequences in the promoter region of target genes^8^. Four W-boxes were found by analyzing the promoter sequence of *ADS* (Supplementary Fig. S3). To test whether AaWRKY17 activates the expression of *ADS* in a direct manner, Y1H and EMSAs were performed. As shown in Fig. 4B, AaWRKY17 bound to two W-box motifs in the promoter region of *ADS* (W2 and W4 boxes). Additionally, the direct binding activity of AaWRKY17 on the W2 box and W4 box in the *ADS* promoter was further verified by EMSA experiments (Fig. 4C, D). These results revealed that AaWRKY17 was a positive regulator of artemisinin biosynthesis by directly activating the expression of *ADS* in *A. annua*.

### The transcriptional expression of *AaWRKY17* is upregulated by *Pseudomonas syringae pv. tomato* DC3000 (*Pst* DC3000) infection and the exogenously applied phytohormones SA and MeJA

**Fig. 5:**
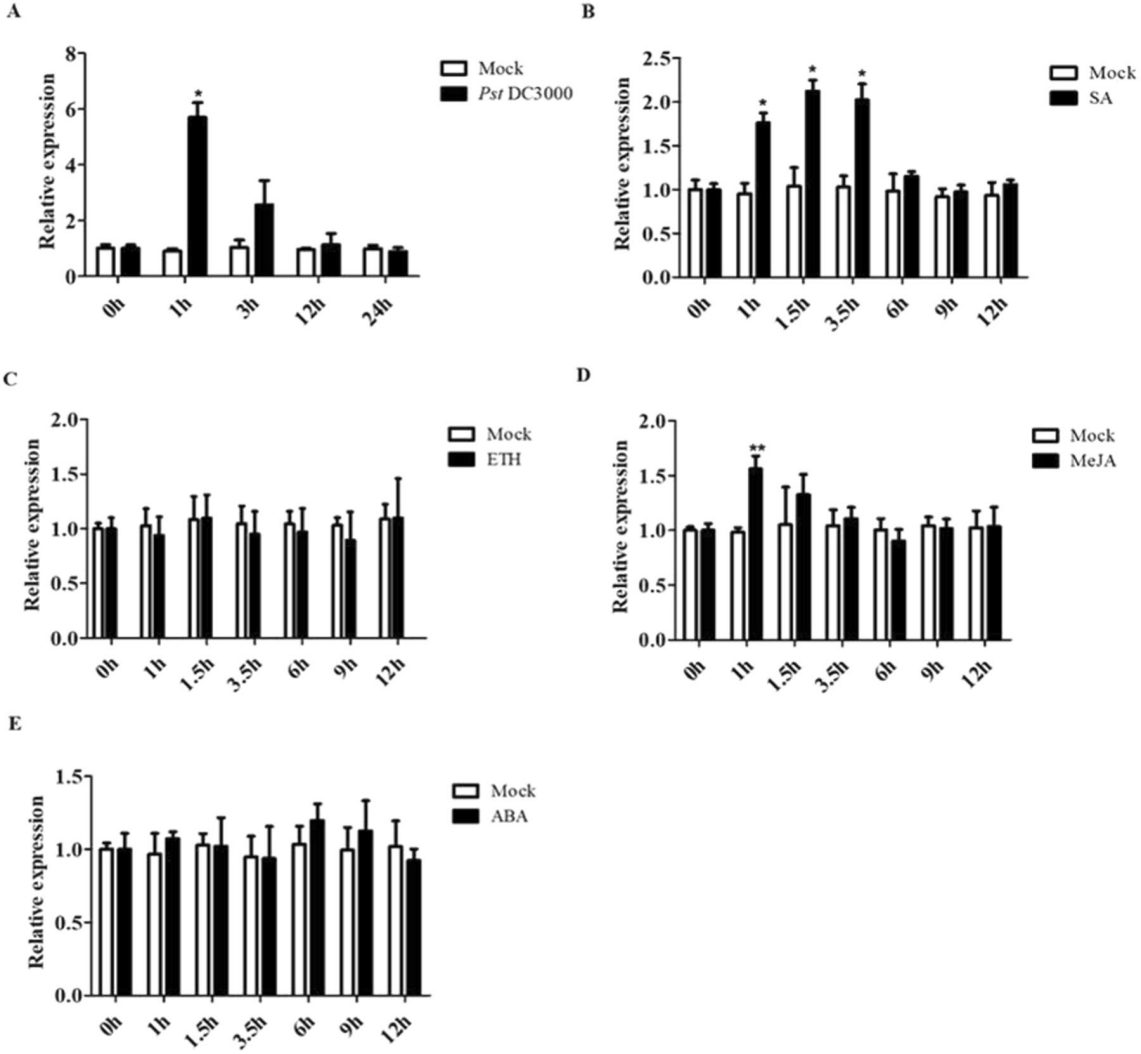
Relative *AaWRKY17* transcriptional levels after *Pst* DC3000 inoculation and different phytohormone treatments. RT-qPCR was performed on samples with **A** *Pst* DC3000 inoculation, **B** application of 1 mM SA, **C** application of 100 μm MeJA, **D** application of 100 μm ETH, and **E** application of 100 μm ABA. **A** Plants were treated with 10 mM MgCl_2_ as a mock treatment. **B**, **D**, **E** Plants were treated with 0.1% ethanol as a mock treatment. **C**, **D** Plants were treated with ddH_2_O as a mock treatment. All data are given as the means ± SD (*n* = 3), *p < 0.05; **p < 0.01; Student’s *t* test

*AaWRKY17* is a homologous gene of *AtWRKY17* that has been proven to be involved in the response to bacterial *P. syringae* in *A. thaliana*. We inferred that AaWRKY17 might also play a role in disease resistance in *A. annua*. To further investigate the possible biological function of AaWRKY17 in plant disease resistance, the expression pattern of *AaWRKY17* was examined after inoculation of WT *A. annua* with *Pst* DC3000. The results of RT-qPCR experiments revealed that the expression of *AaWRKY17* was induced drastically upon *Pst* DC3000 attack compared to that in the mock-treated leaves (Fig. 5A). As shown in Fig. 5A, *AaWRKY17* responded to *Pst* DC3000 quickly and peaked at 1 hpi (hours post inoculation), which implied that AaWRKY17 was potentially involved in the response of *A. annua* to *P. syringae* (Fig. 5A). To further confirm that AaWRKY17 was related to the response to *P. syringae* in *A. annua*, the relative expression of *AaWRKY17* under SA, MeJA, ETH, and ABA treatments was measured by RT-qPCR. The exogenous application of 1 mM SA resulted in significantly enhanced expression of *AaWRKY17* and was maintained until 12 hpt (hours post treatment) (Fig. 5B). Additionally, the transcript level of *AaWRKY17* was also increased when treated with 100 μm MeJA at 1 hpt (Fig. 5D). Compared to the mock treatment, however, the transcript level of *AaWRKY17* showed no obvious difference with the exogenous application of ETH or ABA (Fig. 5C, E). These results indicated that the expression of *AaWRKY17* was greatly induced by *P. syringae* infection and exogenously applied SA and MeJA. Hence, the TF AaWRKY17 was probably involved in the SA and MeJA signaling pathways in response to *P. syringae* in *A. annua*.

### AaWRKY17 functions as a positive regulator of disease resistance to *P. syringae*

**Fig. 6:**
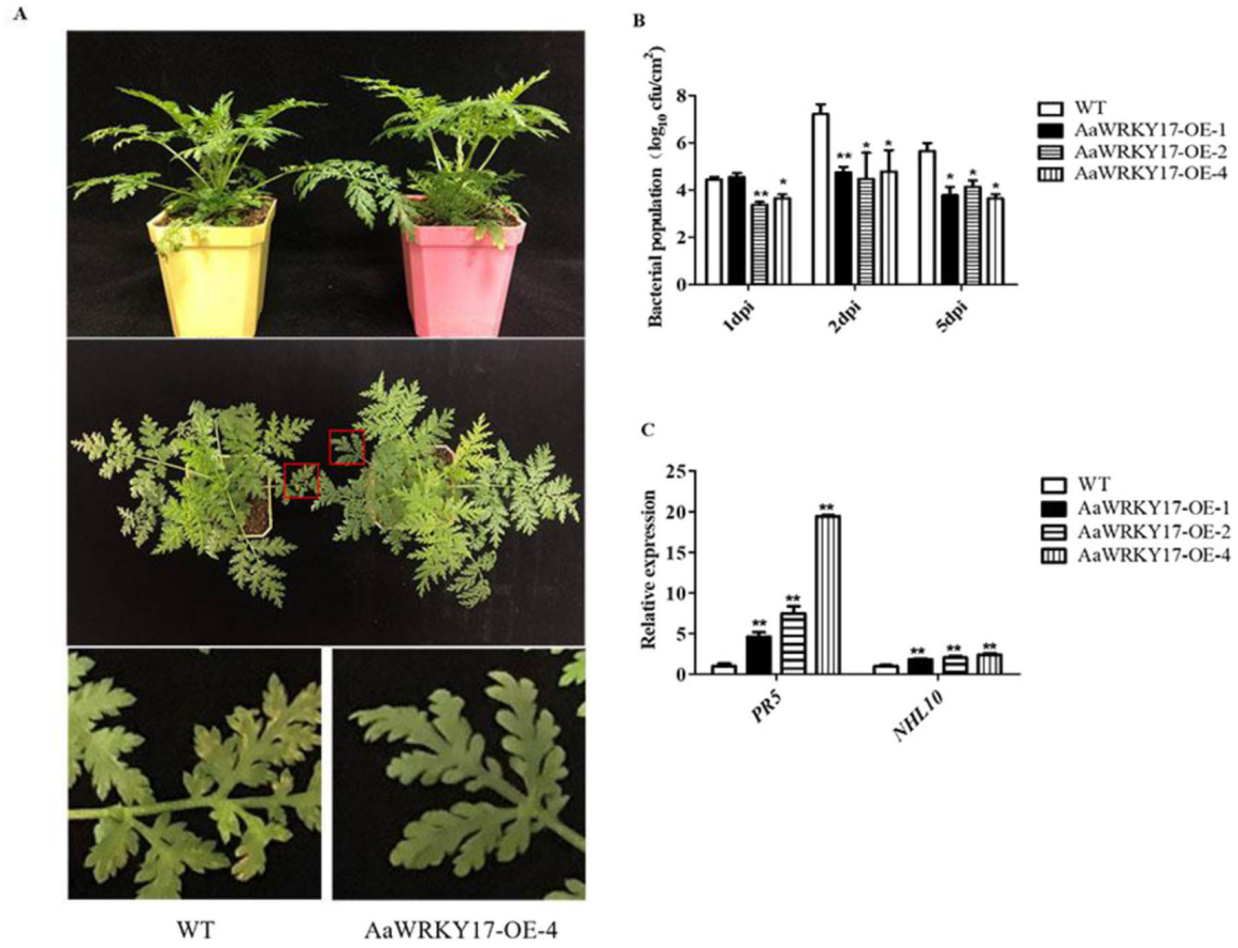
AaWRKY17 functions as a positive regulator of disease resistance to *Pseudomonas syringae*. **A** Phenotypes of WT and *AaWRKY17*-overexpressing transgenic lines at 5 dpi. **B** Bacterial population at 1, 2, and 5 dpi in WT- and *AaWRKY17*-overexpressing transgenic lines. Data are given as the means ± SD (*n* = 3), *p < 0.05; **p < 0.01; Student’s *t* test. cfu colony forming units. **C** RT-qPCR analysis of transcriptional levels of defense-related marker genes in WT and *AaWRKY17*-overexpressing transgenic lines. Data are given as the means ± SD (*n* = 3), *p < 0.05; **p < 0.01; Student’s *t* test

Subsequently, to further test whether AaWRKY17 functions in disease resistance, the phenotypes of *AaWRKY17*-overexpressing lines and WT plants following inoculation with *Pst* DC3000 were observed at 5 dpi (days post inoculation). Compared to the WT plants, disease symptoms were significantly reduced in each of the *AaWRKY17*-overexpressing lines (Fig. 6A). All the leaves of WT plants showed different levels of infection symptoms, whereas only 13% of the leaves from *AaWRKY17*-overexpressing lines were symptomatic. To clearly show the difference in the infected symptoms between WT and *AaWRKY17-*overexpressing lines. We performed a bacterial growth assay with *Pst* DC3000 of infected plants. The statistical analysis results showed that less bacterial growth was observed in *AaWRKY17*-overexpressing plants than in WT plants (Fig. 6B). Moreover, RT-qPCR was used to check the transcriptional expression level of known defense-related marker genes *NHL10* and *PR5*. As shown in Fig. 6C, the expression of *NHL10* and *PR5* was greatly increased in *AaWRKY17*-overexpressing lines (Fig. 6C). These results demonstrated that AaWRKY17 was a positive regulator of disease resistance to *P. syringae* in *A. annua*.

## Discussion

Artemisinin and its derivatives, which are isolated from *A. annua*, have been proven to cure malaria efficiently. In the biosynthesis of artemisinin, ADS catalyzes the conversion of FPP (farnesyl diphosphate) to amorpha-4,11-diene, which is the first step of artemisinin production. Then, amorpha-4,11-diene is converted to dihydroartemisinic acid (DHAA) with the aid of CYP71AV1, DBR2, and ALDH1. After photooxidation, DHAA is transformed to artemisinin in the glandular trichome of *A. annua*. Understanding the transcriptional regulation underlying artemisinin biosynthesis has remained of global concern. In recent decades, numerous TFs, such as those from the TCP^22^, bHLH^23^, bZIP^24^, and WRKY^25,26^ families, have been identified to be involved in artemisinin biosynthesis. In *A. annua*, AaWRKY1 and AaGSW1 are two WRKY TFs that are reported to regulate artemisinin biosynthesis^25,26^. Nevertheless, knowledge about the function of the WRKY family in artemisinin biosynthesis is far from complete. To this end, five WRKY genes that were highly expressed in the trichome of *A. annua* were selected and displayed on a heatmap based on their RPKM values (Fig. 1). Among the five candidate WRKY genes, *AaWRKY17* showed the highest expression in trichomes (Figs. 2A and S1). Therefore, *AaWRKY17* was chosen for generating transgenic *A. annua* plants.

Overexpression of *AaWRKY17* resulted in enhanced artemisinin content, whereas antisense-based gene silencing of *AaWRKY17* decreased artemisinin content in *A. annua* (Fig. 3E, F). In addition, the transcript level of the artemisinin biosynthesis pathway structural gene *ADS* was significantly increased in *AaWRKY17-*overexpressing lines (Fig. 3C, D). To further study the transcriptional regulation of *AaWRKY17*, dual-LUC, Y1H, and EMSA were carried out. As shown in Fig. 5, AaWRKY17 can directly bind to and activate the promoter of *ADS* in vivo and in vitro. Hence, we confirmed that AaWRKY17 was a positive regulator of artemisinin biosynthesis in *A. annua*.

In addition to artemisinin biosynthesis, the role of AaWRKY17 in regulating the biotic stress of *A. annua* was also investigated. Plants are subjected to a variety of biotic stresses during their whole life. Thus, plants have developed a multitude of defense mechanisms to face these stresses, including mechanisms against pathogen stress. As artemisinin is the most promising drug for the treatment of malaria, cancer, and tuberculosis, producing high-yielding *A. annua* is important for meeting the high demand for artemisinin. In contrast to the study of the artemisinin biosynthetic pathway, there is much less information about the effects of different pathogens on *A. annua*. A previous study showed that the trichome-specific AP2/ERF transcription factor AaORA can positively regulate artemisinin biosynthesis and resistance to *B. cinerea* in *A. annua*^34^. Moreover, another AP2/ERF transcription factor, AaERF1, was reported to regulate resistance to *B. cinerea* in *A. annua*^35^. In addition to AP2/ERF TFs, AaNAC1 was found to enhance artemisinin content as well as tolerance to drought and *B. cinerea* in transgenic *A. annua* plants^36^. However, far too little attention has been given to *P. syringae*, which causes economically important diseases in a wide range of plant species. It has previously been observed that the trichomes of plants have a close relationship with disease resistance^37^ and that AtWRKY17 is involved in the response to *P. syringae* in *A. thaliana*^38^. As a homologous gene of *AtWRKY17*, we inferred that *AaWRKY17* may have a similar function in response to *P. syringae* in *A. annua*. RT-qPCR results showed that the transcript levels of the defense marker genes *PR5* and *NHL10* were increased significantly in *AaWRKY17*-overexpressing lines. After inoculation with *Pst* DC3000, compared to the WT plants, most of the *AaWRKY17-*overexpressing lines grew well (Fig. 6). These results demonstrated that AaWRKY17 is a positive regulator of disease resistance to *P. syringae* in *A. annua*.

Additionally, the expression of *AaWRKY17* was induced drastically by the exogenous application of SA and MeJA (Fig. 5). The plant hormones JA and SA have been reported as positive regulators of artemisinin biosynthesis in *A. annua*^39,40^. Furthermore, many pathogen-responsive genes are regulated in an SA- or JA-dependent manner. For instance, WRKY62 was recently reported to have a putative role in modulating the cross-talk between the SA and JA signaling pathways^41^. *A. thaliana* mutant *coi1* showed enhanced susceptibility to *Pst* DC3000 due to the interference of SA synthesis or SA signal transduction, whereas the mutants that were defective in JA signal transduction possessed elevated resistance to *Pst* DC3000^42^. Taken together, our study revealed a new WRKY TF, AaWRKY17, that has dual functions in artemisinin biosynthesis and disease resistance in *A. annua*.

## Materials and methods

### Plant materials

The *A. annua* cultivar used in this study was “Huhao 1”, which had a high artemisinin content of 8–10 mg/g and was originally obtained from the School of Life Sciences, Southwest University, Chongqing^23^. *A. annua* and tobacco *(Nicotiana benthamiana)* were grown under a 16 h photoperiod at 24 ± 2 °C.

### Subcellular localization

For subcellular localization, the ORF of *AaWRKY17* was amplified by PCR and then cloned into the plant expression vector pHB-YFP. The construct was transferred into *A. tumefaciens* strain GV3101 and transiently transformed into 4-week-old tobacco leaves. YFP signals were observed by confocal laser microscopy after 48 h of weak light exposure. Nuclei were stained with 4′,6-diamidino-2-phenylindole staining, and pHB-YFP was used as a negative control. All primers are listed in Supplementary Table S1.

### Hormone treatments

For hormone treatments, 15-day-old WT *A. annua* were sprayed with 100 μM MeJA, 100 μM ETH, 100 μM ABA, and 1 mM SA. For the mock MeJA, ABA, and SA treatment, seedlings were sprayed with 0.1% ethanol. For the mock ETH treatment, the seedlings were sprayed with ddH_2_O. The growth point (GP) and leaf 0 of the seedlings were collected at 0, 1, 1.5, 3.5, 6, 9, and 12 hpt for RT-qPCR analysis. Three biological repeats were performed to verify these results.

### RNA extraction and real-time quantitative polymerase chain reaction (RT-qPCR)

To detect the transcript level of candidate genes in different tissues and leaves at different positions in *A. annua*, tissue samples (stem, old leaves, bud 0, flower, root, shoot, young leaves, and trichome) and leaves at different positions (GP, leaf 0, leaf 1, leaf 2, leaf 3, leaf 4, leaf 5, and leaf 6) were collected as described previously^17^. To analyze the expression of *AaWRKY17*, *ADS*, *PR5*, and *NHL10* in *AaWRKY17* transgenic *A. annua* plants, samples of GP and leaf 0 of a single plant were selected. The total RNA of all samples was extracted using a plant RNA isolation reagent (Tiangen Biotech, Beijing, China), and cDNA was synthesized by using PrimeScript^TM^ RT Master Mix (Takara, Shiga, Japan). Expression levels were normalized to the ACTIN control gene (GenBank accession number EU531837)^39^ and calculated by the 2^−▲▲CT^ method (ref. ^43^). Three independent biological replicates were performed, each containing three technical replicates. All primers are listed in Supplementary Table S1.

### *A. annua* transformation

The constructs pHB-AaWRKY17-YFP, pHB-AaWRKY17-antisense, and 1391Z-*proAaWRKY17*-GUS were introduced into the *Agrobacterium tumefaciens* strain EHA105 and then used to transform *A. annua* as described previously^44^.

### Artemisinin content measurement

Five-month-old *A. annua* leaves were gathered to measure the artemisinin content as described previously^17^. All the *A. annua* leaves were ground to a powder after drying in a 50 °C air oven for 24 h. An aliquot of 0.1 g dry powder was extracted using 1 ml methanol under sonication for 30 min at 55 Hz and then centrifuged for 10 min at 12,000 rpm. The 1 ml supernatant was collected in a new tube. The above steps were repeated, and 2 ml supernatant was collected for the next analysis. The artemisinin content was measured by HPLC^45^. Three biological repeats were measured.

### Dual-luciferase (dual-LUC) assay in tobacco leaves

For the dual-LUC assays, the ORF of *AaWRKY17* was cloned into the pHB vector as an effector, and the promoter of *ADS* was cloned into the pGreenII 0800-LUC vector as a reporter^46^. The effector and reporter were transformed into *A. tumefacien*s strain GV3101 and *A. tumefaciens* strain GV3101, respectively, with the helper plasmid pSoup 19. The pHB empty vector was used as a negative control, and the Renilla luciferase (REN) gene under the control of the constitutive 35S promoter was used as an internal reference. AaMYB17, which was reported not to regulate the expression of *ADS*, was used as a control^47^. Different combinations of the effectors and reporter were mixed in a 9:1 volume ratio to transform 4-week-old tobacco leaves. Leaves were collected after 48 h of incubation under weak light to measure the LUC and REN activities using commercial dual-LUC reaction reagents (Promega). Four biological repeats were performed for each combination. All primers are listed in Supplementary Table S1.

### Yeast one-hybrid (Y1H) assay

Yeast one-hybrid assays were conducted as previously described^26^. The ORFs of *AaWRKY17* were amplified and ligated into the pB42AD vector, and the AaWRKY17 binding sites (W-box) of the *ADS* promoter were cloned into the pLacZ vector. These plasmids were cotransformed into yeast strain EGY48A, which was cultivated on SD/-Ura/-Leu medium for 72 h and tested on SD/-Ura/-Leu medium with 5-bromo-4-chloro-3-indolyl-b-D-galactopyranoside (X-gal) for 48 h. The empty pB42AD and pLacZ plasmids were used as negative controls. All primers are listed in Supplementary Tables S1 and S2.

### Electrophoretic mobility shift assay (EMSA)

For protein expression and purification, the pCold-AaWRKY17 vector was constructed and transformed into *Escherichia coli* strain Rosetta (DE3) (TransGen Biotech, China). Heterologous protein production was induced by adding 0.5 mM isopropyl β-D-1-thiogalactopyranoside to the bacterial culture for 16 h at 16 °C and purified using HisSep Ni-NTA (nitrilotriacetic) agarose resin (Yeasen, China). The W2-box, W4-box, mW2-box, and mW4-box probes from the promoter of *ADS* were synthesized and then labeled with biotin at their 5′ end by Sangon (Shanghai, China). EMSAs were performed using the LightShift Chemiluminescent EMSA Kit (Thermo, USA) following the manufacturer’s instructions. The probes used in the EMSA are listed in Supplementary Table S2.

### *Pseudomonas syringae pv. tomato* DC3000 (*Pst* DC3000) inoculation and quantification

Six-week-old *A. annua* plants were used for bacterial inoculation. Briefly, *Pst* DC3000 was cultivated at 28 °C and 200 rpm in King’s medium B with rifampicin (50 mg/l), collected by centrifugation, resuspended in 10 mM MgCl_2_ at OD_600_ = 0.6 and supplemented with 0.04% Silwet L-77 for spray inoculation^48^. After *Pst* DC3000 inoculation, the plants were kept at 100% relative humidity. The GP and leaf 0 were sampled at 0, 1, 3, 6, 12, and 24 hpi for RT-qPCR. For quantification of bacterial populations, six leaves (10 mm^2^ × 6) from one individual plant were collected at 1, 2, and 5 dpi, washed twice with sterile water and homogenized in King’s B containing rifampicin, followed by 10^−1^, 10^−2^, 10^−3^, 10^−4^, 10^−5^, and 10^−6^ dilutions on solid medium.

## Supplementary information

supplementary
