# Supplementary material for: AaWRKY17, a positive regulator of artemisinin biosynthesis, is involved in resistance to Pseudomonas syringae in Artemisia annua
Source: Hortic Res. 2021 Oct 1;8:217. doi: 10.1038/s41438-021-00652-6 (PMC8484609; doi:10.1038/s41438-021-00652-6)

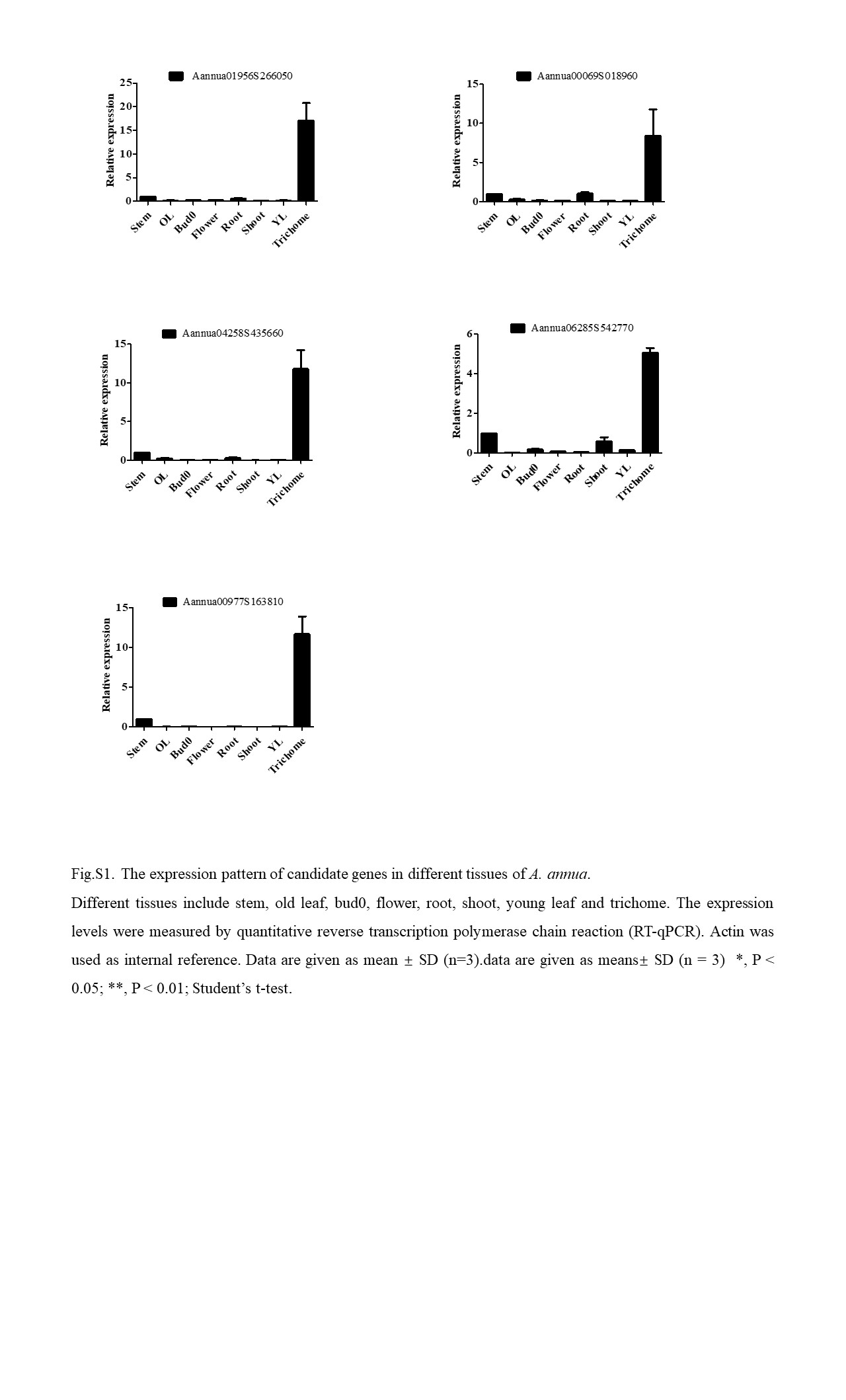


Fig.S1. The expression pattern of candidate genes in different tissues of *A. annua.*

Different tissues include stem, old leaf, bud0, flower, root, shoot, young leaf and trichome. The expression levels were measured by quantitative reverse transcription polymerase chain reaction (RT-qPCR). Actin was used as internal reference. Data are given as mean ± SD (n=3).data are given as means ± SD (n = 3).


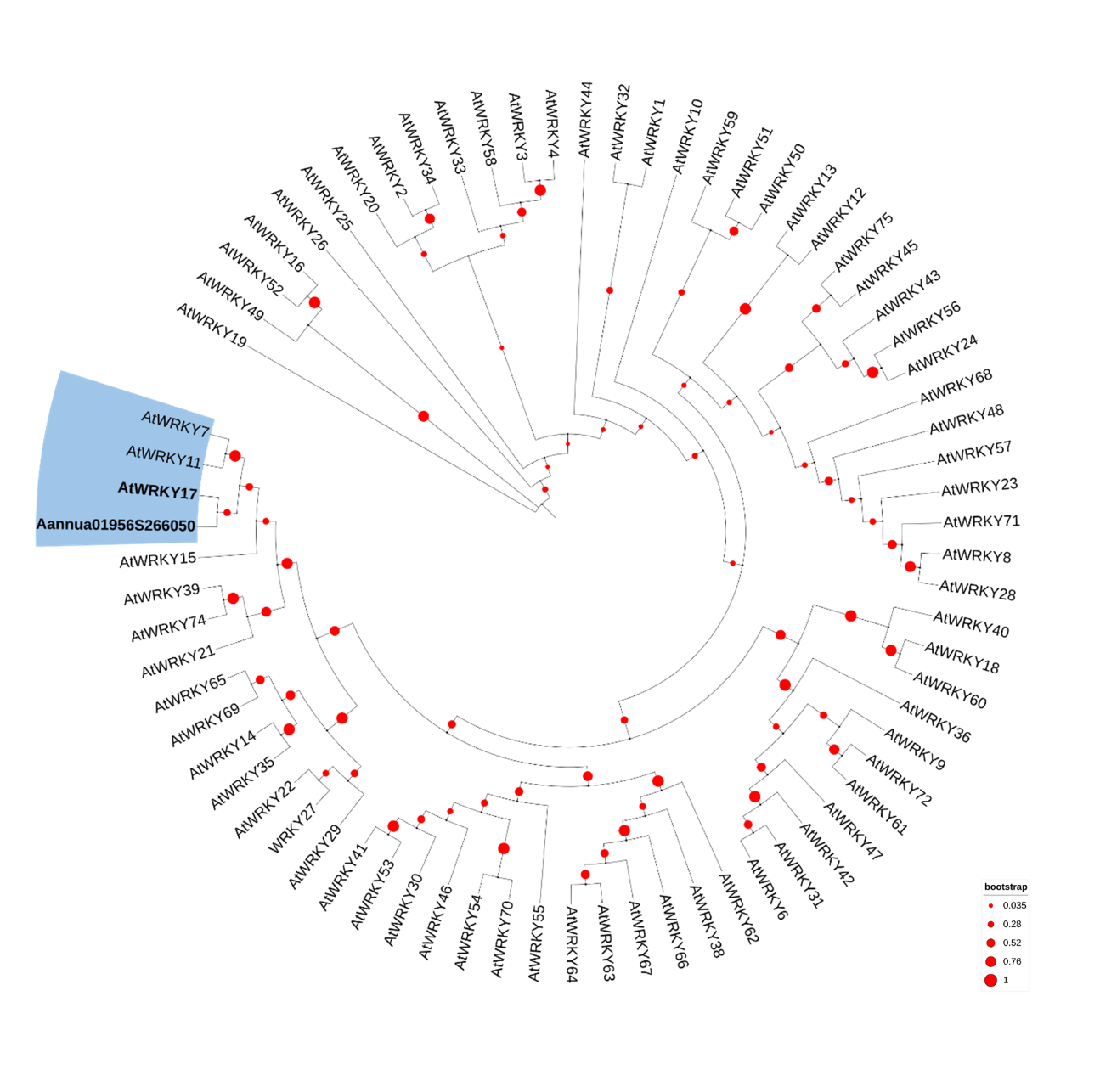


Fig.S2. Phylogenetic tree showing the relationship between the candidate *A. annua* WRKY transcription factor Aannua01956S266050 and all WRKY transcription factors from *Arabidopsis*


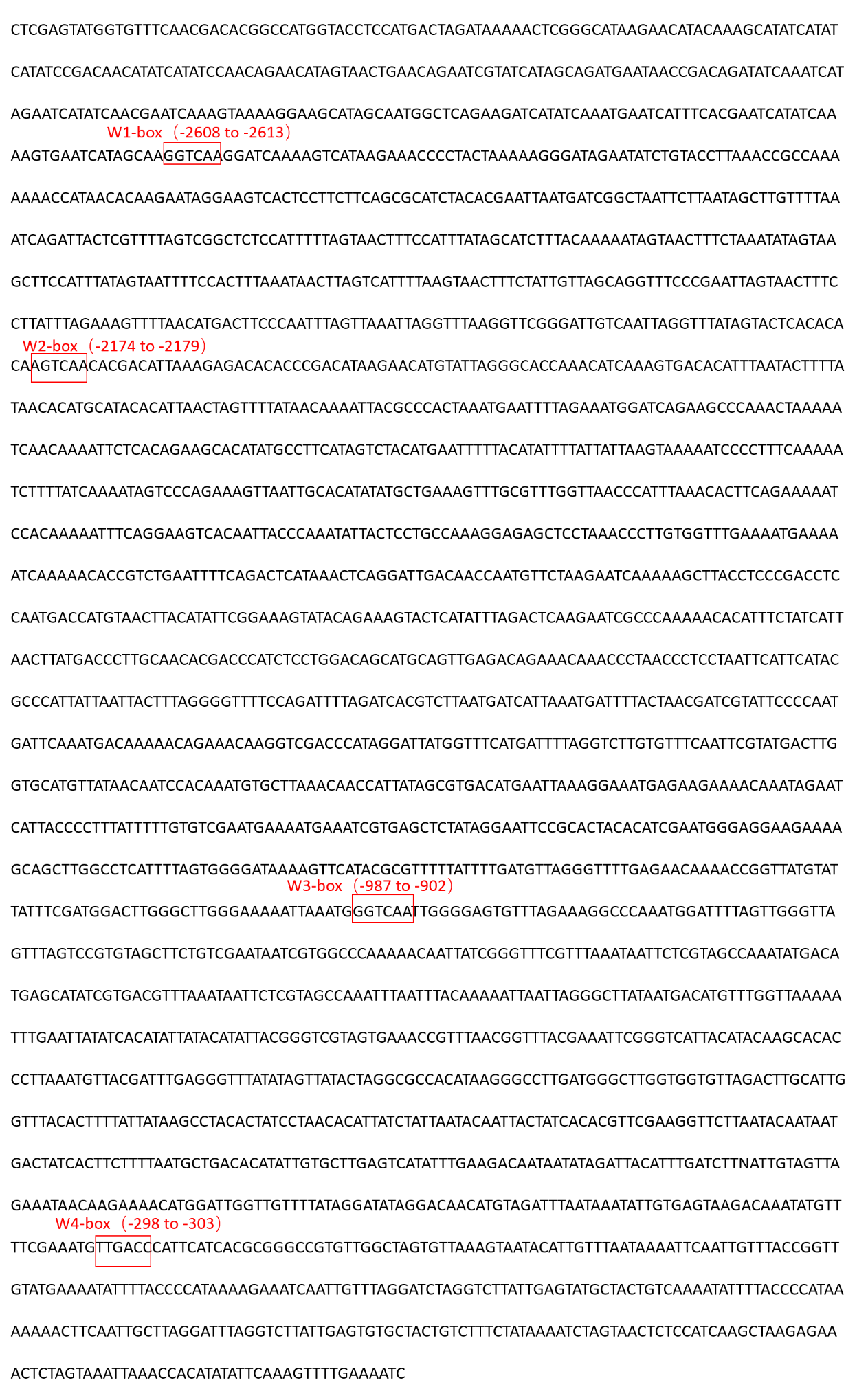


Fig.S3. Promoter sequence of ADS

Table S1. All primers used in this study


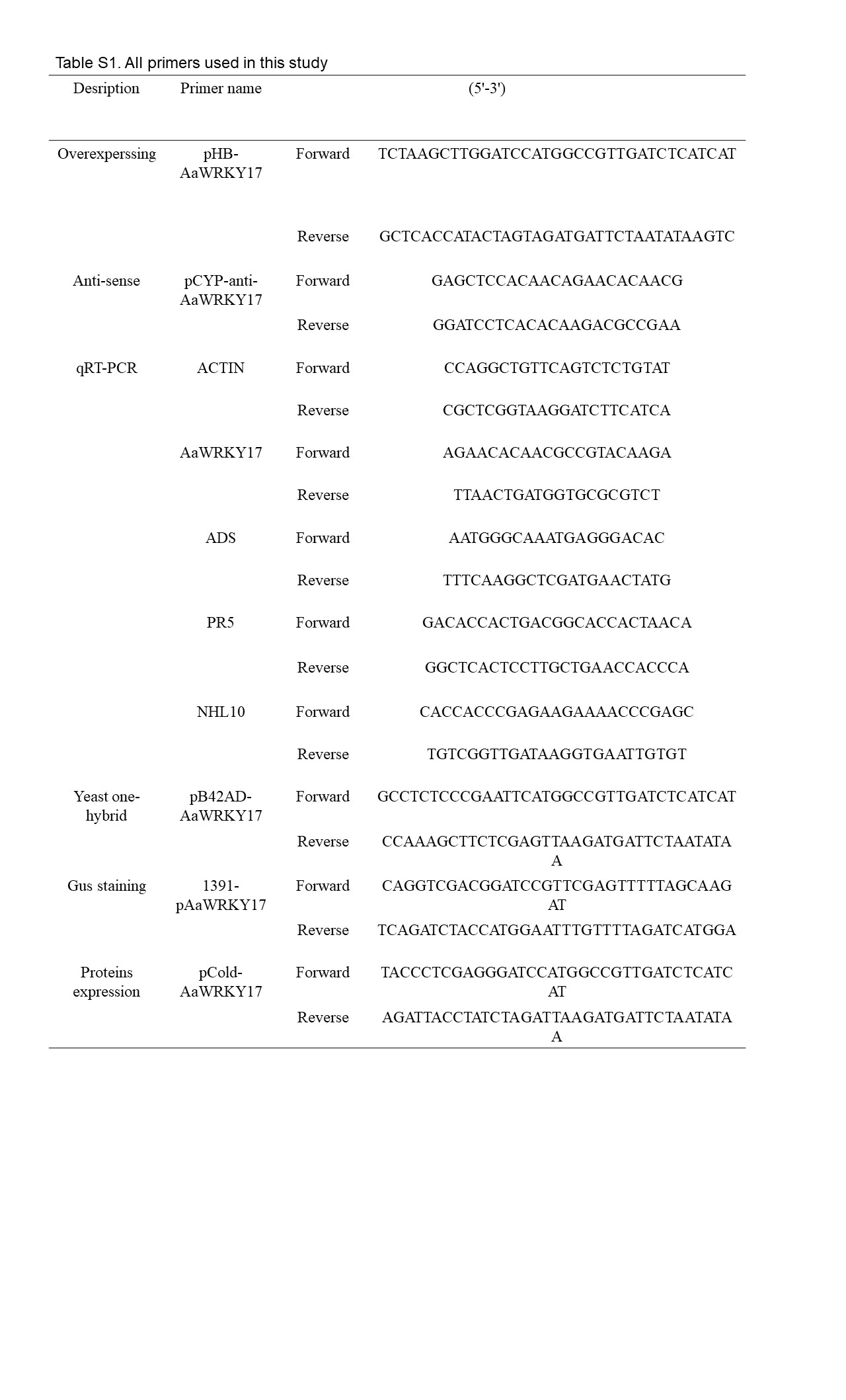


Table S2. Fragments used to construct yeast one-hybrid vectors and as probe in EMSA in this study


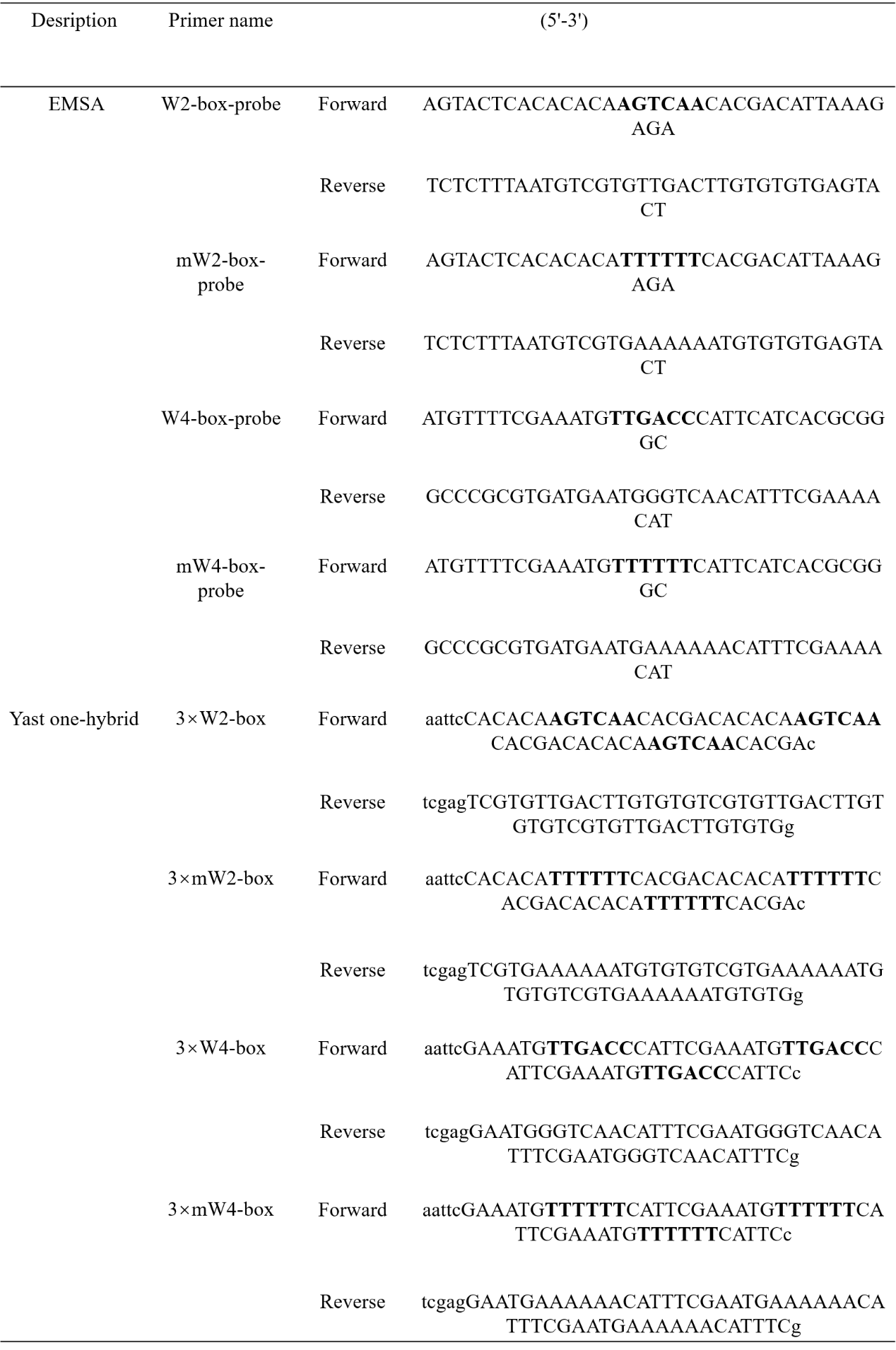

Supplement: Supplementary file 1 — supplementary [file 41438_2021_652_MOESM1_ESM.docx]
